# Memory Load Influences Taste Sensitivities

**DOI:** 10.3389/fpsyg.2018.02533

**Published:** 2018-12-11

**Authors:** Pei Liang, Jiayu Jiang, Qingguo Ding, Xiaoyan Tang, Soumyajit Roy

**Affiliations:** ^1^Department of Psychology/Facuty of Education, Hubei University, Hubei, China; ^2^Brain and Cognition Research Center (BCRC), Faculty of Education, Hubei Univeristy, Hubei, China; ^3^The No. 2 Peoples’ Hospital of Changshu, Changshu, China; ^4^Changshu Institute of Technology, Changshu, China; ^5^Eco-Friendly Applied Materials Laboratory, Materials Science Centre, Department of Chemical Sciences, Indian Institute of Science Education and Research, Kolkata, India

**Keywords:** cross-modal, memory load, cognitive status, sweetness perception, bitterness perception

## Abstract

Previous literature reports have demonstrated that taste perception would be influenced by different internal brain status or external environment stimulation. Although there are different hypotheses about the cross-modal interactive process, it still remains unclear as of how the brain modulates and processes taste perception, particularly with different memory load. Here in this study we address this question. To do so we assign the participants different memory loads in the form of varying lengths of alphanumerical items, before tasting different concentrations of sweet or bitter tastants. After tasting they were asked to recall the alphanumerical items they were assigned. Our results show that the memory load reduces sweet and bitter taste sensitivities, from sub-threshold level to high concentration. Higher the memory load, less is the taste sensitivity. The study has extended our previous results and supports our previous hypothesis that the cognitive status, such as the general stress of memory load, influences sensory perception.

## Introduction

In today’s fast paced society, on one side, many people have quick meals “on the go.” Their cognitive brain is still busily processing something related with work while chewing and swallowing meals. It has been suggested that cognitive load would distract the attention and reduce sensory perception. Several studies have demonstrated that taste perception may be influenced by internal brain state such as attention and awareness (Elder and Krishna, 2009). The literature has been very mixed regarding the influence’s effect between attention and multi-sensory integration (Odegaard et al., 2016). For instance, some investigations have found that attention has no effect on multisensory integration (Bertelson et al., 2000; Shore and Simic, 2005), while other studies have reported that selective attention can increase integration (Alsius et al., 2005), or reduce integration (Mozolic et al., 2008; Talsma et al., 2010). Yantis (2000) has proposed the competition of limited attention resources. When attention is distracted by other information resources like TVs, the taste perception would get less sensitive and the hedonic rating of the food would be reduced. Similarly, people experience tastants less intense when the environment has loud noise (Spence and Shankar, 2010). Stafford et al. (2012, 2013) have demonstrated that music and distraction may alter taste perception of alcohol. How the brain processes the sensory perception, particularly involved with the neural network of higher level of cognition, remains unclear. Kahneman (2011) has proposed two cognitive processes. Accordingly, the cognitive states are divided into “cognitive ease” and “cognitive strain.” The cognitive ease means people feel effortless and comfortable. When the brain is at cognitive ease state, the response is fast and intuitive. On the contrary, when the brain is at cognitive strain state, people feel less comfortable or stressed. It leads people to increase attention and to invest more effort and the corresponding response is more critical. Our previous studies have demonstrated that visual information affects taste sensitivities (Liang et al., 2013, 2016). For instance, the visual stimuli representing color, shapes, or symbols induce attentive mechanisms, which are difficult to calibrate with respect to the cognitive ease and load concept. And such components might contribute differently to affecting the gustatory perception. Motivated by Kahneman’s theory and the previous research, we hypothesized that the cognitive status plays a key role in gustatory perception. More specifically, we would like to focus this study on cognitive strain and observe how taste perception thresholds are affected by a simple memory load task.

Recently van der Wal and van Dillen (2013) have tested whether task load influences the sweet, sour, and salty perception. In their study they demonstrated that the task load reduces not only aversive tastants, such as sourness of lemon juice, but also pleasant tastants like grenadine syrup and salty butter. However, in their study, the tastants are more complex, and the tastants concentrations are relatively higher compared with taste threshold level. It would be therefore important to observe how the basic taste sensitivity changes at lower concentrations to avoid the saturation of the taste receptors or central habituation. Hence, our present paper is to study how the sweetness and bitterness sensitivity is influenced with different degrees of memory load, particularly, at low concentration level (around taste threshold).

To test if cognitive strain influences the perception thresholds, we manipulated the degree of memory load by varying the length of alphanumerical items. Two basic but hedonically opposite tastes, i.e., sweetness and bitterness were applied and calibrated against the memory load. The results confirm the hypothesis that cognitive load reduces taste perception.

## Materials and Methods

### Subjects

Twenty-six student volunteers (sixteen females and ten males) from Changshu Institute of Technology (CIT), China were chosen for the experiments. They were all self-reported right-handed and had normal eyesight or at least were corrected to normal by glasses. None of them was color blind and their ages were between 21 and 30 years old (average 25 ± 3 years). They did not have any taste- or smell-related disease before. All the participants were well briefed about the details of the experiments and of their performance. They all agreed and signed on the written informed consent declaration to volunteer as subjects in these experiments. The study was approved by the Ethics Committee (IEC) of the No. 2 Peoples’ Hospital of Changshu (license number 20151101), according to the Ethics Guidelines.

### Tastants Preparation

We applied sucrose solution as sweet tastants. Sucrose was dissolved in distilled water to prepare the sugar solution with concentration of 0, 1.5, 3.1, 3.9, 4.7, and 5.5 g/L, respectively (Liang et al., 2013). The phenylthiourea solution was prepared with concentration of 0, 0.02, 0.04, 0.08, 0.16, and 0.24 mM/L, respectively. All the sweet and bitter solutions were prepared one night before and kept on the table at the room temperature between 20 and 25°C. During the experiments the solutions were provided to the subjects in a series of half-filled odorless white paper cups (25 ml).

### Memory Load on Display

**FIGURE 1:**
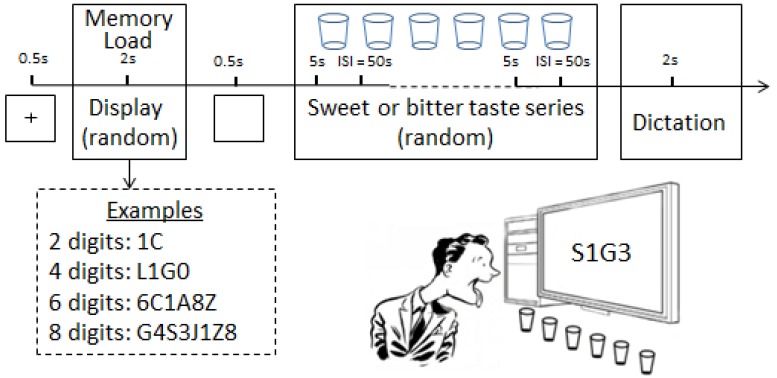
The upper part represents the time schematics of experiment flow. “+”is displayed on the center of monitor for 0.5 s at the beginning of each trial. The below left side describes the examples of lists of alphanumerical items, which consist of alphabets and numbers in random order and are displayed on the center of the monitor in front of the subjects. Six cups represent the six different concentrations of sweet or bitter tastants. ISI means the 50 s rest for the subjects after tasting each cup. In the end of the whole blocks, the subject has 2 s to recall the list of alphanumerical items. The below right side depicts the experiment setup.

Before tasting experiments, the participants were visually exposed to a list of alphanumerical items on the monitor for 2 s. They were requested to remember and to repeat the information given to them after each block of taste experiments (refer to Figure 1 Experiment flow). The memory load task was modified by changing the length of the list of alphanumerical items. For example, “1C,” “L1G0,” “6C1A8Z,” “G4S3J1Z8” represent the four groups of memory tasks with increasing cognitive load. The number and alphabet are in a staggered random arrangement without any possibility of association and remembrance by the volunteers, during repetition of the experiments. The information of memory task was displayed on 17-inch LCD Monitor with 60 Hz refresh rate.

### Experiment Training

The experiments are carried out at the sensory science laboratory, Changshu Institute of Technology, Jiangsu, China. There are two purpose of the experiment training: (1) to pre-test the sweet and bitter taste threshold level and (2) to confirm that tastants concentrations are suitable for the experiments. To measure the sweetness or bitterness threshold, the subjects performed the sweet or bitter taste series of six different concentrations randomly without being exposed to visual stimuli. The sweetness and bitterness thresholds were measured by the staircase method and forced choice tracking procedures (refer to the Pepino and Mennella, 2010). The participants were trained for 2–4 times to perform the memory load task and taste experiments, till they were confident and comfortable with the experiments. To avoid the influence of hunger status of the subjects, the experimental data of the subjects were collected at a fixed time of the day (around 1 h after food intake) while repeating the experiments on different days.

### Procedures

During the tasting experiments, the subject sat in front of the table. Six cups of sugar solution with different concentrations were placed on the table next to the subject. Before tasting experiments, the monitor would display the list of alphanumerical items, i.e., randomly generated numbers and alphabets, for 2 s. The tasting experiments followed. For the sugar solution experiments, the subjects sipped the sugar solution from the paper cup (around 12 ml) into the mouth and moved the tip of the tongue slightly, keeping the solution in the mouth for 5 s and spitted it out. During the following 50 s pause, the subjects rinsed mouth twice with distilled water, answered the questionnaire whether they detected sweet taste from the corresponding solution. The bitter taste experiments were carried out similarly. The subjects needed to finish all the different taste concentrations in the sweet or bitter taste blocks. We used “1,” “-1” and “0” to record the results of the taste experiment. When the participant detected the sweetness from the solution, “1” is recorded. When participant detected bitterness from the solution, “-1” is recorded. If the participant did not detect any sweet nor bitter taste, “0” is recorded. In the block experiment, the sweet or bitter solutions of different concentrations were provided to the subjects in random order, respectively. The participants were requested to recall the visual information after each trial. The recall feedbacks would not be reported to the participants. And the next trial will restart after 2 s. The memory task with different length of digital inputs for the subjects was generated randomly in a complete block design. Every participant needed to perform the whole set of the blocks and the trials were repeated ten times. All the experiments were carried out at room temperature 20–25°C.

### Data Analysis

All the data were recorded and saved in the computer (Window system 7) and were analyzed offline with MATLAB 7.9 (The MathWorks, Natick, MA, United States). We calculated *the sweetness or bitterness detection ratio* of each memory load for each person. *The taste detection ratio* = *the number of times when sweet or bitter taste detected with each memory task*/*the number of times of total experiments repeated with the corresponding task*. For each memory load, the taste detection ratio of each tastants concentration is first analyzed per person. The average detection ratio and the standard deviation across 26 persons are calculated accordingly. For each concentration of sweet or bitter taste, the within group one way repeated ANOVA was used to test the significance of differences in the taste detection ratios with different memory load. And the *post hoc* test Bonferroni adjusted was used to perform the pairwise comparison within the group.

## Results

**FIGURE 2:**
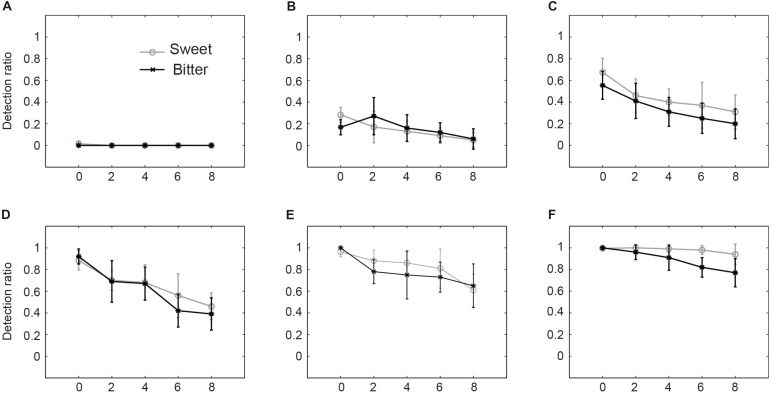
Both sweet and bitter taste detections decrease with memory load. **(A–F)** Present the sweetness detection with sucrose concentration of 0, 1.5, 3.1, 3.9, 4.7, and 5.5 g/L, and bitterness detection with phenylthiourea concentration of 0, 0.02, 0.04, 0.08, 0.16, and 0.24 mM, respectively. The gray circles and black stars represent the sweet and bitter taste detection ratios averaged across all the subjects and the error bars denote the standard deviations across all the subjects. *Y*-axis represents the sweetness and bitterness detection ratio, from 0 to 1, 1 represents 100 percent detection. *X*-axis represents the length of the lists of alphanumerical items as the memory load during taste experiments, 0 represents the detection ratio without memory task.

With manipulation of different degrees of memory load (five types), we tested the sweet and bitter taste detection of participants with a series of different concentrations of sugar and phenylthiourea (each taste includes six groups). The results show that when the memory load increases, both the sweet and bitter taste detection ratios decrease significantly (detail data refer to Tables 1, 2). During the training phase, the threshold of sweetness was found between 3.1 and 3.9 g/L, and bitterness threshold between 0.04 and 0.08 mM of phenylthiourea solution. These data were similar to our previous lab observations (Liang et al., 2013). For the taste experiments with cognitive load, 2 (Tastants: Sweet vs. bitter taste) × 6 (Groups: six different concentrations for each tastants) × 5 (Types: five different lengths of memory load tasks) within-group repeated ANOVAs were done separately for the six groups of each tastants, and the levels compared pairwise in *post hoc* tests. The averaged taste detection ratios of different concentrations of both sweet and bitter taste are illustrated in Figure 2, and supplemented by Tables 1, 2.

**Table 1:** Sweetness detection ratios of different sugar concentration under variant memory load.

	Solution concentration					
Task load	0 g/L	1.5 g/L	3.1 g/L	3.9 g/L	4.7 g/L	5.5 g/L
0	0.01 ± 0.02	0.24 ± 0.10	0.58 ± 0.20	0.89 ± 0.10	0.95 ± 0.06	1.00 ± 0.01
2	0.00 ± 0.00	0.13 ± 0.14	0.49 ± 0.18	0.73 ± 0.12	0.88 ± 0.09	1.00 ± 0.00
4	0.00 ± 0.00	0.11 ± 0.09	0.44 ± 0.16	0.68 ± 0.14	0.84 ± 0.11	0.99 ± 0.03
6	0.00 ± 0.00	0.09 ± 0.08	0.33 ± 0.22	0.57 ± 0.21	0.83 ± 0.17	0.98 ± 0.04
8	0.00 ± 0.00	0.06 ± 0.08	0.27 ± 0.15	0.52 ± 0.16	0.69 ± 0.19	0.95 ± 0.08


*Horizontal list labels represent the sugar concentration and the vertical list labels the length of alphanumerical memory load.*

**Table 2:** Bitterness detection ratios of different concentration under variant memory load.

	Solution concentration					
Task load	0 mM	0.02 mM	0.04 mM	0.08 mM	0.16 mM	0.24 mM
0	0.00 ± 0.00	0.18 ± 0.07	0.55 ± 0.11	0.93 ± 0.66	1.00 ± 0.00	1.00 ± 0.00
2	0.00 ± 0.00	0.26 ± 0.16	0.39 ± 0.16	0.70 ± 0.18	0.80 ± 0.12	0.96 ± 0.06
4	0.00 ± 0.00	0.16 ± 0.11	0.31 ± 0.12	0.68 ± 0.15	0.77 ± 0.22	0.92 ± 0.11
6	0.00 ± 0.00	0.13 ± 0.09	0.25 ± 0.13	0.46 ± 0.20	0.75 ± 0.15	0.84 ± 0.10
8	0.00 ± 0.00	0.09 ± 0.13	0.24 ± 0.17	0.43 ± 0.18	0.68 ± 0.21	0.79 ± 0.14


*Horizontal list label represent the phenylthiourea concentration and the vertical labels the length of alphanumerical memory load.*

With the still water, as expected, almost no taste was detected with whatever memory load (Figure 2A). At very low concentration, the detection ratio decreases significantly with increasing memory load (Figure 2B, gray line). Although the averaged bitterness detection has similar tendency as the sweetness detection line, no significant effect has been found (Figure 2B, black line). Repeated ANOVA result shows that when the bitter solution concentration is at 0.04 mM, the bitterness detection ratio decreases significantly with increasing memory load [Figure 2C, black line, *F*(4,105) = 17.070, *p* < 0.001]. When bitter taste concentration got even higher (above the threshold level), the decreasing trend of taste detection ratio became more obvious [Figures 2D,E, black lines, D: *F*(4,105) = 33.041, *p* < 0.001; E: *F*(4,105) = 11.498, *p* < 0.001]. Repeated ANOVA results shows that when sweet solution is 3.1 g/L, the sweetness detection ratio decreases significantly with increasing memory load [Figure 2C, gray line, *F*(4,125) = 1.774, *p* < 0.001]. It is worth to note that Figure 2D represents the maximal decrease of both sweetness and bitterness taste detection ratio from around 90% (no memory load) to around 40% (maximal memory load) [sweetness: *F*(4,125) = 22.058, *p* < 0.001]. At highest taste concentration of our experiments (sugar 5.5 g/L), the sweetness detection was all close to 100% [Figure 2F, gray line, *F*(4,125) = 4.664, *p* < 0.01]. This may be explained by the sweetness response saturation. However, the bitterness detection ratio still reduces when the memory load increases [Figure 2F, black line, *F*(4,105) = 17.635, *p* < 0.001]. It should be noted that the sweetness and bitterness concentrations are differently prepared. We would expect that at very high bitterness concentration, we might observe similar saturation effect as the sweetness perception.

## Discussion

This study has systematically tested the changes of sweetness and bitterness sensitivities with different degrees of memory load. The sweetness and bitterness sensitivities both decrease significantly when memory load increases. These results are consistent along the lines of the hypothesis, that cognitive status influences the taste perception. Our previous publications (Liang et al., 2013, 2016) have shown that the cognitive ease induced by visual inputs such as circular shapes, familiar words may enhance the sweetness sensitivity. The cognitive strain induced by angular shapes, unfamiliar words may reduce the sweetness perception. The cognitive ease or strain induced by the visual shapes or words in the previous studies is mostly referred to affective or hedonic cognition, i.e., cognitive positive and ease or cognitive negative and strain. The stimuli were passive and unfocused for the participants. Different from the previous paper, this study has applied the phonological loop as memory task, which request the participants to repetitively recall the visual alphanumerical information. These task leads active and focused attention of the participants and make them distracted from the taste itself, and thus reduces the taste perception. Moreover, our observation is consistent with other literature that the task load influences the sweet, sour, and salty perception, where much complex and higher concentration tastants have been studied (van der Wal and van Dillen, 2013). All these studies are consistent with the observation that task load, specifically negative or strain load, reduces the taste perception.

Different from the common food in the market with usually relatively high taste concentration, the low concentration of tastants are not much studied. This study has extended the previous literature finding and studied the sweet and bitter taste from sub-threshold to beyond threshold level. When the tastant concentration is extremely low (close to zero), there is no significant effect of memory load on taste perception (Figure 2B). When the tastants concentrations increase to around threshold level, the effect of memory load becomes more prominent (Figure 2C). As the tastant concentration increases and is more beyond the threshold level, we still observe the significant effect of memory load. Both sweetness and bitterness sensitivities decrease with increasing memory load (Figures 2D,E). It is worth to mention that the taste concentrations applied in our experiments are far below the market food or drinks (Huang et al., 2018). The reason is that we are focused to observe the taste perception around the taste threshold level, which has been suggested in our previous paper (Liang et al., 2013, 2016), the uncertain zone in our brain perception around threshold level is easier to be influenced by external stimuli. Marks and Wheeler (1998) have found that the thresholds are lower for attended tastants of sucrose and are critical than unattended ones. However, the attention in their study is limited to the expectation by giving subject the cue of tastants. In our study we extend the investigation to the more general memory load, such as food irrelevant memory load, and observe how are the sweet- and bitter- taste sensitivities influenced by the memory load.

Regarding the internal brain status induced in our experiments, the active and attentive process was generated by memory task, which would lead the subjects to cognitive strain. Under such cognitive state, the sensitivity of the subjects toward taste detection has been observed to be reduced. Higher the cognitive strain, lesser is the sensitivity toward the taste. Recently van der Wal and van Dillen (2013) have demonstrated similar observation that task load reduces sweet, salt, and sour taste perception. In their experiments, the cognitive load was to instruct the subjects to remember seven-digit number (high load) or one-digit number (low load). Such induced brain load is affective neutral and non-food related. Similarly, the task applied in our study was also without affective bias, helped us to observe the effect in a more general and systematic pattern.

Several previous studies have examined the effect of distracting stimuli on food choice (Shiv and Nowlis, 2004; Nowlis and Shiv, 2005). Earlier research shows due to cognitive load the taste perception reduces, and thus people tend to have more food to retain the same preferred taste levels in an attempt to preserve the enjoyment level of the food as compared to relaxed food intake conditions. On the other side, the cognitive status induced by emotion (negative or positive) may influence the taste perception as well. Noel and Dando (2015) found that sour taste was enhanced with negative emotion. The brain mechanisms of taste perception under different cognitive states remain unclear. When the attention of the subject is focused on taste pleasantness, the medial orbitofrontal and pregenual cingulate cortex are greater active than when attention is instructed to taste intensity. The taste detection in a tasteless solution involves insula and overlying operculum (Veldhuizen et al., 2007). Such finding might indirectly support our hypothesis that variant cognitive status affects taste perception differently.

Moreover, our data in this study may be explained from evolutionary biology (Wilson, 2014). We note that gustatory perception of taste sensitivity as that of bitterness is related to survival (Wooding, 2005) and has lower threshold (than sweetness for instance). When cognitive load is applied, cognitive processing takes precedence and gustatory perception sensitivity may reduce, even with bitterness sensitivity which is related to survival (Diamond, 1998). This might be explained by that cognitive beings survived due to cognitive calculations and cautionary steps taken rather than gustatory explorations. Hence when under cognitive stress the gustatory sensory processing manifest as bitterness taste sensitivity takes a backseat registering a decrease in bitterness taste sensitivity. It further leads us to another factor that influences gustatory perception, the affective component. We relate cognitive ease status to this affective component in the matter of gustatory perception. Previously, we have shown that visual information from external environment such as shapes influences taste sensitivities (Liang et al., 2013). These experiments explored the affective aspect of gustatory taste perception. The circular shapes inducing cognitive ease (positive emotion) were shown to enhance the sweetness sensitivity. The sweetness detection in our previous experiment is more associated with affective positive and cognitive ease stimuli. The bitterness detection as described in this experiment is more associated with cognitive strain stimuli (Kahneman, 2011). The present experiment shows when under cognitive stress the gustatory sensory processing manifest as bitterness/sweetness taste sensitivity takes a backseat registering a decrease in bitterness taste sensitivity. Hence it is reasonable to speculate that the influence of cognitive over the affective in the context of gustatory sensory processing would be a weighted average of both affective and cognitive components. However, how affective and attentive cognitive components associate with each other and influence gustatory perception is not possible to shed light on here; although this may be an interesting topic for future studies with our calibrated model.

Here we infer that the different cognitive (for instance, attentive) states might be the key factor, which contribute to the modification of sweetness and bitterness perceptions. This study show that the memory load influences both sweet and bitter taste in a similar pattern (Figures 2B–E). Both taste detections reduce dramatically when the memory load increases. In our experiments, only the neutral cognitive load was applied, no affective component was induced, thus, the sweet and bitter taste sensitivities were influenced similarly. On the other hand, in our previous experiments, the visual inputs have both affective and cognitive components and where affective components were also allowed to exercise their influence alone by the choice of the sweet as the only tastants.

Although this study has extended the task stimuli from our previous studies, still the phonological loop as memory load here is a specific task, which involves silent repetition of verbal information coded from visual information (the list of items displayed on the monitor). The observation is limited to support completely the hypothesis of the study. To reach a more general cognitive load effect, it might be useful to design the articulation suppression paradigm with this material, or to use a non-verbal type of material in future. Moreover, regarding the potential difference of sweetness and bitterness perception from the evolutionary aspect, one should be aware that there may be an evolutionary twist to the human sugar intake. Fructose may be considered natural to us, and might be interesting to test the effect of fructose in our future experiments.

## Conclusion

To conclude, the current work has demonstrated that the sweetness and bitterness detection ratios decrease with increasing memory load, especially around threshold concentration. At higher concentration, the both taste detection ratios are unaffected by the memory load. It is consistent with previous observation from other laboratories and extends our understanding to a more systematic pattern. Higher the memory load, lesser is the taste sensitivities. This study supports the hypothesis that the cognitive states (positive-ease or negative-strain) influences taste perception, and which of course still has a long way to go before we understand it completely. On a lighter note our work suggests that stress and enjoyment of food do not gel well with each other, and the cognitive process induced by different eating life style may modify the taste perception and lead to the acquired taste.

## Author Contributions

PL and SR designed the experiments and wrote the manuscript. JJ and QD performed the experiments. PL, JJ, and XT performed the data analysis.

## Conflict of Interest Statement

The authors declare that the research was conducted in the absence of any commercial or financial relationships that could be construed as a potential conflict of interest.
